# Policy dialogue as a collaborative tool for multistakeholder health governance: a scoping study

**DOI:** 10.1136/bmjgh-2019-002161

**Published:** 2020-04-17

**Authors:** Emilie Robert, Dheepa Rajan, Kira Koch, Alyssa Muggleworth Weaver, Denis Porignon, Valery Ridde

**Affiliations:** 1 Institut universitaire SHERPA, CIUSSS du Centre-Ouest-de-l'Île-de-Montréal, Montreal, Quebec, Canada; 2 World Health Organization, Geneva, Switzerland; 3 CEPED (French Centre for Population and Development), IRD (French Research Institute for Development) (IRD–Paris Descartes University), Paris, France

**Keywords:** health systems evaluation, health policy, health systems, review

## Abstract

**Introduction:**

Health system governance is the cornerstone of performant, equitable and sustainable health systems aiming towards universal health coverage. Global health actors have increasingly been using policy dialogue (PD) as a governance tool to engage with both state and non-state stakeholders. Despite attempts to frame PD practices, it remains a catch-all term for both health systems professionals and researchers.

**Method:**

We conducted a scoping study on PD. We identified 25 articles published in English between 1985 and 2017 and 10 grey literature publications. The analysis was guided by the following questions: (1) How do the authors define PD? (2) What do we learn about PD practices and implementation factors? (3) What are the specificities of PD in low-income and middle-income countries?

**Results:**

The analysis highlighted three definitions of policy dialogue: a knowledge exchange and translation platform, a mode of governance and an instrument for negotiating international development aid. Success factors include the participants’ continued and sustained engagement throughout all the relevant stages, their ability to make a constructive contribution to the discussions while being truly representative of their organisation and their high interest and stake in the subject. Prerequisites to ensuring that participants remained engaged were a clear process, a shared understanding of the goals at all levels of the PD and a PD approach consistent with the PD objective. In the context of development aid, the main challenges lie in the balance of power between stakeholders, the organisational or technical capacity of recipient country stakeholders to drive or contribute effectively to the PD processes and the increasingly technocratic nature of PD.

**Conclusion:**

PD requires a high level of collaborative governance expertise and needs constant, although not necessarily high, financial support. These conditions are crucial to make it a real driver of health system reform in countries’ paths towards universal health coverage.

**Key questions**

**What is already known?**

- Health system governance is an overlooked area which needs strengthening in countries’ path towards universal health coverage.
- Collaborative mechanisms such as policy dialogue are emerging as a key facilitating factor for strengthening multistakeholder health governance.
- The concept is characterised by inconsistent definitions, stakeholders’ hazy understanding of the concept and the challenge of evaluating its implications.

**What are the new findings?**

- Policy dialogue may be understood as a knowledge exchange and translation platform, a mode of governance or a negotiating instrument in international development.
- Policy dialogue, as a multistakeholder collaborative governance tool, requires critical skills from both facilitators and participants, as well as adequate and sustained funding.
- The following conditions are necessary to foster continued stakeholder engagement in policy dialogue: a transparent and institutionalised policy dialogue process, a shared understanding of the goals of policy dialogue and a policy dialogue approach that fits the intended goals.

**What do the new findings imply?**

- Because of limited country-level organisational and technical capacities in low-income and middle-income countries, skills in the realm of health system governance should be fostered.
- There is a need to step up efforts to build the capacity of stakeholders for and support policy dialogue as a valuable health system governance tool.
- Policy dialogue processes and activities require steady and predictable monies rather than substantial financial support, as well as a high level of technical expertise.

## Introduction

Governance involves ‘ensuring strategic policy frameworks exist and are combined with effective oversight, coalition-building, regulation, attention to system-design and accountability’.^1^ It is ‘a process of coordinating stakeholders, social groups and institutions to achieve objectives that have been collectively defined and discussed’ (Le Galès, p 301)^2^. In the health sector, governance is the cornerstone of performant, equitable and sustainable health systems aiming towards universal health coverage (UHC).^3 4^

Health system governance builds on the engagement of a range of different stakeholders, from within the health sector^5^ and beyond.^6 7^ These stakeholders contribute, directly or indirectly, to putting in place and implementing public standards, strategies and policies. In low-income and middle-income countries (LMIC) and aid-dependent settings, health system governance has long been overlooked, as urgent and critical health challenges were prioritised by global health actors and governments.^8^ The recent epidemic of Ebola shed light on the devastating health effects of weak governance.^9^ UHC, which initially focused on health financing,^10^ became a window of opportunity to strengthen health system governance.^4 11^

As a complex coordinating process, health system governance requires fine-tuned skills in bringing diverse views together, brokering and consensus-building. Policy dialogue (PD) has recently emerged as a promising governance tool to enhance the quality of engagement between state and non-state stakeholders,^12^ and to address the health sector’s cross-cutting challenges.^13^ It provides an awaited opportunity for collaboration, particularly to contribute to Sustainable Development Goals. These goals lay specific emphasis on involving a diversity of stakeholders, including civil society and the population, from various sectors in policy-making, with a view to more participatory governance.^14^ PD could thus help solve current cross-cutting and multisectoral challenges,^15^ such as UHC.^11^

By facilitating the inclusion of civil society^16^ and bilateral and multilateral development partners, PD would be an enabling tool to support the co-development of measures that support health targets.^17^ It is seen as a means of contributing to ‘robust’ and ‘realistic’ policies^18^ in that they are informed by evidence.^19 20^ PD also responds to commitments made in the Accra Agenda for Action^21^ on enhancing the effectiveness of development aid and is favoured by some development agencies as a negotiating instrument for international cooperation.^22 23^

In this context, PD raises considerable expectations. Yet, researchers and professionals face two main challenges when dealing with PD. First, PD remains a vague concept,^24^ both for those who are involved in it and those organising or supporting it. Guidance on best practices, recommendations and empirical studies are therefore useful.^12 19 25–28^ However, this merely highlights the plethora of definitions of PD. Second, there is dire evidence on the effectiveness of PD. A recently published journal supplement on PD sought to evaluate its impact,^12^ examining PD based on the definition put forward by the World Health Assembly in 2011:

‘an inclusive policy dialogue with a comprehensive range of stakeholders, within and beyond government, including civil society organisations, the private sector, and health professionals and academics, within the health and other sectors, is critical to increasing the likelihood that national policies, strategies and plans will be appropriately designed and implemented and will yield the expected results’ (p. 18).^18^

This definition uses epidemiological terminology and presupposes that it is possible to measure the impact of PD. The challenge, therefore, is to operationalise collaboration, negotiation and decision-making processes, which are known to be complex.^29^ Evaluating PD is even more arduous as such processes eventually lead to outcomes that are not predictable.

We sought to clarify the concept of PD. Here, we identify the component elements, success factors and challenges of PD by reviewing the literature on the subject. This work is part of an evaluation of the UHC Partnership,^17^ a WHO programme to support Ministries of Health to build capacity for PD as a critical missing link on the path to UHC.^30^

## Methods

### Research questions

This literature review took the form of a scoping study,^31^ which involved a systematic examination of the literature on PD and analysis based on the following questions:

- How do the authors define PD?
- What do we learn about PD practices, as well as the social, political, organisational, institutional and other factors that influence PD?
- What are the specificities of PD in LMIC?

### Search strategy

First, a review of the literature conducted by the first author identified relevant papers in the Medline, CINHAL, PubMed, Web of Science and Scopus databases up to September 2017. Table 1 lists the databases, the keywords and the number of articles in each search. Four criteria were used to select the relevant literature to address the questions mentioned above:

**Table 1:** Details of databases consulted and searches

Databases	Searches	Number of articles
Medline	(in TITLE)	Total=204
(including CAB Abstracts—1910–2016 week 47; Embase 1974–2016 week 49; Global Health 1910–2016 week 47; all OvidMedline 1946–present)	policy AND dialogue	After automatically deleting duplicates=132
		After manually deleting duplicates=126
Medline	(in TITLE)	Total=49
(including CAB Abstracts—1910–2017 week 36; Embase 1974–2017 week 38; Global Health 1910–2017 week 36; all OvidMedline 1946–present)	‘stakeholder dialogue’ OR ‘multi-stakeholder dialogue’ OR ‘multistakeholder dialogue’	After automatically deleting duplicates=32
		After manually deleting duplicates=23
CINHAL	(in TITLE)	Total=81
(including Anthropology+International Political Science Abstracts+Public Administration Abstracts+Social sciences abstracts+remove Medline citations)	policy AND dialogue	After automatically deleting duplicates=75
		After manually deleting duplicates=74
PubMed	(in TITLE)	Total=44
	policy AND dialogue	
Web of Science	(in TITLE)	Total=227
	policy AND dialogue	
Scopus	TITLE (‘policy dialogue’)	Total=365
		Primary documents=74
		Secondary documents=291
		After automatically deleting duplicates=331
Total		Total=802
		After automatically deleting duplicates=731
		After manually deleting duplicates=**592**

- Criterion 1: the article is in English.
- Criterion 2: the article is published in a peer-reviewed journal.
- Criterion 3: the article focuses on PD as a decision-making process involving state and non-state players in national arenas, whether in low-income, middle-income or high-income countries, or international arenas. We excluded other forms of multi-stakeholder engagement,^32^ such as public consultation processes.
- Criterion 4: the article reports empirical research, or conceptual or theoretical thoughts, or a review of the literature on PD. We excluded articles merely describing sets of PD activities.

Two additional stages of documentary research followed. In the first one, papers quoting the articles from the initial search were searched using Web of Science. Then, the list of references for each of the articles was examined to identify articles on PD. In the second stage, grey literature identified by WHO to develop a concept note on PD was consulted.^19^ Selection criteria 1 and 3 were applied to these papers. We adjusted criterion 4 to reflect that grey literature (ie, documents published by public, commercial or industrial entities) may not report robust empirical research, but rather lessons learnt. Criterion 4 for grey literature was labelled as follows: “The article reports lessons learnt or review of the literature on PD. We excluded articles merely describing sets of PD activities”.

We did not solely search for experiences of PD in the health sector, but instead looked for insights from multiple sectors (eg, agriculture, water, etc) included in various disciplinary journals. We aimed to broaden the scope of the literature review while highlighting the cross-cutting challenges of conducting PD.

### Analysis

Peer-reviewed papers were analysed by the first author, with NVivo qualitative data analysis software using an inductive and thematic approach. The themes that were identified formed a framework for analysing the papers that emerged from the subsequent stages. Grey literature was also analysed by the first author, with the support of three of the coauthors.

### Patient and public involvement

It was not appropriate to involve patients or the public in the design, conduct, reporting or dissemination plans of our research.

## Results

### Description of the articles included

**Figure 1:**
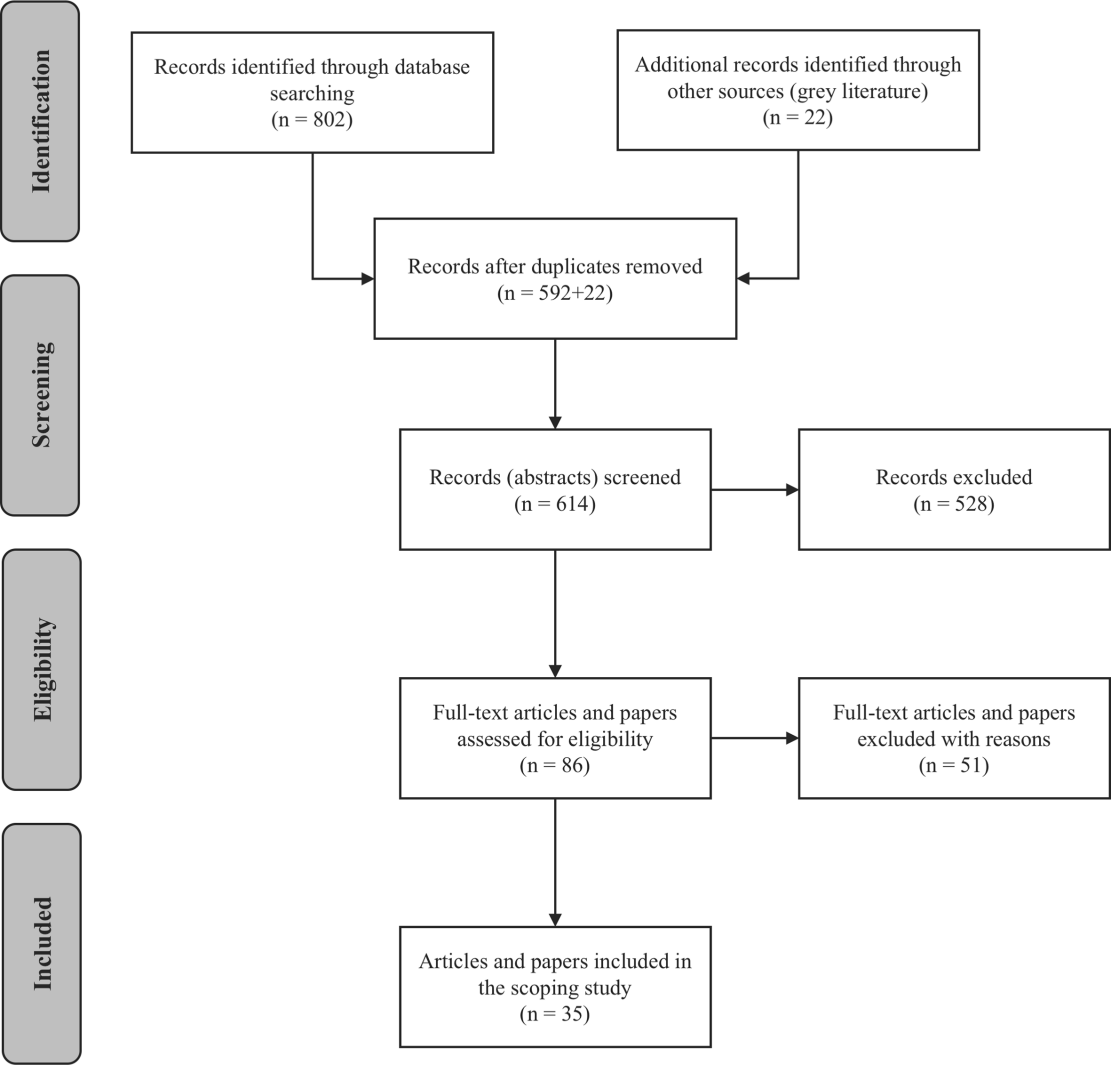
Process of selecting articles and documents (Preferred Reporting Items for Systematic Reviews and Meta-Analyses diagram).

The total number of papers included was 35 (figure 1). Phase I of the research identified 592 papers from peer-reviewed literature, of which 25 articles met the selection criteria. Phase II identified 21 papers from grey literature, 10 of which met the selection criteria. In total, 25 were on PD in the context of LMIC. The two fields most represented were health (n=12) and development aid (n=13). Other fields included the environment (n=2), agriculture (n=3), migration (n=1), transport (n=1), water (n=1), social protection (n=1) and urban planning (n=1). Most of the papers were empirical studies (n=12) or case studies and comments (n=11), where evidence is usually qualitative. Table 2 lists the papers and clarifies their method and objective.

**Table 2:** Description of the documents in the scoping study

Study	Year	Geographical area*	Field†	Method‡	Objective
**CONCEPTUAL LITERATURE**					
Ehrmann and Lesnick^62^	1986	N/A	N/A	N/A	Describe the main aspects and best practice on PD in policy development.
Lavis *et al* 20	2010	N/A	Health	N/A	Propose questions to guide the organisation and implementation of health policy dialogue.
Nabyonga-Orem *et al* 12	2016	AFRO	Health	N/A	Take stock of the health policy dialogue in low-income African countries.
**EMPIRICAL STUDIES**					
Ade *et al* 42	2016	AFRO	Health	QUAL	Review the coordination of PD in the context of Ebola virus disease in Guinea, as perceived by the participants.
Boyko *et al* 33	2016	HIC	Health	MIXED	Explore the characteristics and outcomes of a deliberative public health policy dialogue on family violence in Canada.
Damani *et al* 34	2016	HIC	Health	MIXED	Review the contribution of PD to the development of an evidence-based policy on access to care in Canada.
Dovlo *et al* 43	2016	AFRO	Health	QUAL	Explore PD in the development of three policies in Cape Verde, Mali and Chad.
Jagger *et al* 44	2014	LMIC	Environment	QUAL	Understand how environmental protection policy dialogue at different levels (international, national, local) influences the development of national policies.
Kwamie and Nabyonga-Orem^39^	2016	AFRO	Health	QUAL	Understand how health policy dialogue can contribute to aligning stakeholders in Guinea and Chad.
Lavis *et al* 35	2014	HIC	Health	QUANT	Evaluate three health policy dialogues in Canada.
Mwisongo *et al* 46	2016	AFRO	Health	QUAL	Understand the power issues underlying PD in Chad, Cape Verde, Guinea, Liberia and Togo.
Nabyonga-Orem *et al* 61	2016	AFRO	Health	QUAL	Examine what PD means for stakeholders in Cape Verde, Chad, Guinea, Togo and Liberia.
Nabyonga-Orem *et al* 45	2016	AFRO	Health	QUAL	Understand how context influences health policy dialogue, including stakeholder participation in Liberia.
Scholten and Verbeek^40^	2015	EURO	Migration	QUAL	Understand how the politicisation of the issue of migration may influence the institutionalisation of related PD.
Uneke *et al* 36	2015	AFRO	Health	QUANT	Evaluate PD for infectious disease control in Nigeria.
**CASE STUDIES AND COMMENTS**					
Babu and Ergeneman^38^	2005	ASIA	Agriculture	N/A	Document the interaction between knowledge producers and decision-makers as part of agricultural policy dialogue in India.
Bangura^81^	1997	N/A	Development aid	N/A	Discuss PD and its implications with respect to gender equity and participation.
Baumann and White^41^	2014	EURO	Transport	N/A	Understand how and under what conditions a transport policy dialogue in Munich enabled stakeholders to overcome their disagreements.
Biswas^72^	2004	INT	Water	N/A	Discuss the progress of international discussions on water and how it has been influenced by the format of PD.
Daniel^53^	1985	LMIC	Development aid	N/A	Identify the relevance of PD to development aid and the conditions for it to succeed.
Dhunpath and Paterson^47^	2004	LMIC	Communication	N/A	Discuss the participation of researchers in PD on information and communication technology in Botswana, Namibia and Seychelles.
Drechsel *et al* 37	2013	AFRO	Agriculture	N/A	Describe the process of irrigation policy dialogue in Ghana and capacity-building efforts for participation in this PD.
Goldsmith^60^	2016	ASIA	Agriculture/Development aid	N/A	Describe the agricultural policy dialogue in India and identify lessons learnt for development aid.
Molenaers^51^	2012	LMIC	Development aid	N/A	Discuss PD as a platform for negotiating budget support.
Susskind and Hoben^48^	2004	HIC	City	N/A	Discuss the obstacles and possible solutions for municipal PD.
White^50^	1990	AFRO	Development aid	N/A	Highlight the limitations of technocratic approach to PD in development aid.
**GREY LITERATURE**					
AusAID^54^	2011	N/A	Development aid	N/A	Propose a theory of policy dialogue success.
Brown *et al* 56	2012	ASIA	Social protection/Development aid	QUAL	Identify the factors behind the successful PD on social protection in Indonesia.
Brown *et al* 55	2012	ASIA	Development aid	QUAL	Identify the factors behind the successful PD framework for cooperation on financial and economic reform in the Solomon Islands.
Canadian International Development Agency^57^	2002	N/A	Development aid	N/A	Identify lessons learnt on PD from the Canadian development aid agency.
Crouch^49^	1993	N/A	Development aid	N/A	Identify lessons learnt on PD to initiate a dialogue and drive forward reforms.
Jacob and Alvarado^58^	2011	N/A	Development aid	N/A	Assist development agencies in their support for PD on education.
Jones^59^	2011	N/A	Development aid	N/A	Facilitate development agency engagement in PD.
McCullough *et al* 28	2011	N/A	Development aid	N/A	Suggest key elements to evaluate PD between donors and recipient countries.
The SURE Collaboration^27^	2011	N/A	N/A	N/A	Propose guidelines to organise deliberative PD.
Visser and Adey^32^	2007	N/A	Environment	N/A	Explain the relevance of PD launched by entrepreneurs to tackle climate challenges.

*N/A/AFRO/HIC/LMIC/ASIA/EURO/INT.

†N/A.

‡N/A/QUAL/QUANT/MIXED.

AFRO, country(ies) located in the African continent; ASIA, country(ies) located in the Asian continent; EURO, country(ies) located in the European continent; HIC, high-income country(ies); INT, multiple countries in different continents and with different incomes; LMIC, low-income and middle-income country(ies); MIXED, mixed study; N/A, not applicable; QUAL, qualitative study; QUANT, quantitative study.

### A polysemous concept

The literature highlights three notions of PD (table 3): a knowledge exchange and translation platform, a mode of governance and a negotiating instrument.

**Table 3:** Classification of the documents in the scoping study

Knowledge exchange and translation platform (n=8)	Mode of governance (n=14)	Negotiating instrument (n=14)
**Empirical literature**		
Boyko *et al* 33 Damani *et al* 34 Lavis *et al* 35 Uneke *et al* 36	Ade *et al* 42 Dovlo *et al* 43 Jagger *et al* 44 Kwamie and Nabyonga-Orem^39^ Mwisongo *et al* 46 Nabyonga-Orem *et al* 61 Nabyonga-Orem *et al* 45 Scholten and Verbeek^40^	Daniel^53^ Goldsmith^60^ Molenaers^51^ White^50^
**Conceptual/Theoretical literature**		
Lavis *et al* 20	Ehrmann and Lesnick^62^ Nabyonga-Orem *et al* 12	Bangura^81^
**Case studies/Comments**		
Babu and Ergeneman^38^ Drechsel *et al* 37	Baumann and White^41^ Biswas^72^ Dhunpath and Paterson^47^ Susskind and Hoben^48^	
**Grey literature**		
The SURE Collaboration^27^		AusAid^54^ Brown *et al* 56 Brown *et al* 55 Canadian International Development Agency^57^ Crouch^49^ Jacob and Alvarado^58^ Jones^59^ McCullough *et al* 28 Visser and Adey^32^

As a knowledge exchange and translation platform, PD aims to support evidence-based decision-making.^20 27 33–38^ This approach is based on knowledge translation theories, such as those elaborated on by Lavis *et al*.^20^ PD is understood to consist of well-prepared, organised and structured interactions (meetings, events, etc) between diverse stakeholders (researchers, civil society, decision-makers, etc), often initiated by the research community to translate evidence into policy-making. It is deliberative in that an emphasis is placed on the balanced presentation of evidence with ample room for reflection; the format of the interaction is a critical part of PD. In that regard, PD is considered the end of a process involving preliminary steps to prepare the activity.

As a mode of governance, PD aims to formulate a plan, strategy or policy in a participatory manner. Therefore, a decided focus is given to the exchange of knowledge, joint drafting of documents and facilitated debate among stakeholders to find common ground or compromise. This dialogue is generally initiated by government authorities at national^39–47^ or even local level.^41 48^ With this approach, the evidence provides the base material around which informing exchanges occur. The PD takes the form of a series of formal or informal meetings which culminate in one or more major events—forums, workshops, meetings, etc—where stakeholders come together to discuss the issues at hand.

As a negotiating instrument, PD provides a means of influencing governments on policy. This approach differs from PD as a mode of governance in that it is generally initiated or encouraged by non-state actors, such as development partners or lobbies,^49^ with a strategic objective. There are two types of literature on PD as a negotiating instrument. The first is scientific literature, generally from the 1990s, and is drawn from the discipline of development studies.^50–53^ Here, PD is an instrument for negotiation between international agencies and partner countries on donor-supported development projects and policies. The second is grey literature, which is published or commissioned by international organisations,^28 54–59^ and in which PD is seen as an entry point to participate in and influence country-level public policy in the sectors targeted by development aid.

### The component elements of policy dialogue

Regardless of the approach, and whether it relates to a specific activity or a (longer) process, PD appears to consist of a few fundamental elements that ensure its implementation:

1. The literature points to a clear definition of the PDs objectives and topic as critical to ensuring a well-tailored PD methodology.^27 35 43 45^ A well-formulated stated objective also shapes the participants’ expectations and the overall expected outcomes.^33 35^
2. The issue of who feels free to speak and who speaks up seems critical. Discussion formats for smaller groups (which are not necessarily open to the general public) are thus widely discussed in the literature,^27 41^ as are the necessity for Chatham House rules.^33 35 36^ Sociocultural aspects would also influence who speaks when and how much time is given for each person to speak.^45 46^
3. Funding for PD appears necessary, particularly in low-income countries.^39 42^ Both financial and logistical support would ensure the soundness and longevity of PD.^55 56^ For stakeholders to perceive PD as fair and legitimate, funding should come from a neutral source.^41^
4. Reliable evidence is the foundation on which discussions are based.^33 39 59^ Some authors point to the necessity of high-quality, contextualised, credible and relevant evidence to be available before PD^20 50 60^ to ensure that discussions are meaningful.^45 61^
5. The convener and facilitator roles look critical for successful PD. The convener should have the organisational capacity to conduct the PD, which may be problematic in low-income countries where institutions do not always have the necessary resources and skills.^42 43^ The facilitator^35 42 48^ should combine the skills of a mediator and technical expertise,^27 33 39 43^ and create an environment that is conducive to building consensus,^48^ while simultaneously demonstrating neutrality and impartiality.^41^
6. The participation of all relevant stakeholders would ensure the process is fair and legitimate.^41^ In contrast, failure to include certain groups, such as the private sector^35^ or the community,^45^ could undermine the credibility of the process. The choice of participants can also be strategic as some players may hinder the implementation of the proposed solutions.^48^
7. It is also essential to consider the participants’ capacity to analyse, summarise and criticise so that they can make a constructive contribution to the exchanges.^27 58 61^ The participants should have sound knowledge of the institutional and political context, both to guide the PD process and possible outcomes and to propose viable courses of action.^27 56^ Finally, they would need to understand their own organisation’s position on the issue being discussed and be able to speak on its behalf.^27^ This may prove difficult in the context of development aid as experts from international organisations are often inextricably tied to internationally agreed recommendations,^53^ limiting the contribution they can make. Lack of experience and expertise could lead to a power imbalance between the participants.^46 48 50 60^ Pre-PD training would, therefore, be useful.^37 46 53^ In low-income countries, these issues need to be examined further in light of the challenge of finding enough skilled and available human resources to engage in processes that are often time-consuming and where staff turnover may be high.^43 60 62^

### Factors that influence policy dialogue

The literature highlights three factors: (a) the organisational context, (b) the political and institutional context and (c) power relations, particularly in the context of development aid.

Concerning the organisational context, participating organisation’s values would influence both the openness towards and the engagement in PD of their representatives, hence influencing PD outcomes. Representatives may hinder discussions or organisational procedures of PD unless their organisation values or is open to PD, conveys the idea that such a process is in their interest and does not fear that other participants challenge its authority.^43 48^ In the context of development aid, the standpoint of international organisations are said to affect the nature and quality of participation: some would be more inclined to contribute and participate as part of their mandate^53^ or when the PD topic is on their agenda.^28 53 56^ The position that participants occupy within their organisation may also influence PD. The choice of representative and their position in the hierarchy are indicators of an organisation’s level of commitment and interest in PD.^41^ PD initiated at a senior level can mobilise suitable participants and ensure that colleagues who represent them have authority or expertise.^42 43 45^ However, there may also be resistance from institutional stakeholders seeking a leadership role.^56^ Some invitation protocols may increase the number of participants; any disadvantages should be measured against the expected outcomes.^20 27^

Concerning the political and institutional context, Jones highlights five factors that influence PD and its results in the context of development aid^59^: (1) the level of power separation; (2) regulation and competition between political players; (3) the relationship between country governments and external development partners; (4) stakeholder capacity to absorb change and (5) ‘informal’ policy dynamics. In other words, each country’s different approaches to politics shapes its institutional context within which PD happens.^59^ One such approach to politics can be categorised as ‘neopatriotic’, where the hierarchical position within state structures matters less than the individuals’ personal proximity to power; this would create a bias in PD that includes institutional representatives.^46^

In addition to these factors, some of the literature refers to windows of opportunity^33^ as periods when stakeholders are more open to change.^38^ These moments should be leveraged for engaging stakeholders, encouraging an evidence-informed approach to decision-making^20^ and facilitating political negotiations.^60^ However, the increasingly technocratic nature of PD may imply that the political economy of the decision-making process is given insufficient attention by technician-experts who are unable to identify such windows of opportunity.^50 51 59^ The literature describes several bilateral donor-recipient PD processes that failed due to the international technician-experts’ lack of understanding of the country’s political context.^50 51 59^

Concerning the balance of power between PD participants, most of the literature here is within an aid-dependent country context.^46^ The first challenge is the perception of the donor’s agenda being the main stimulus for PD, thereby hindering the ownership of the topic at the heart of the PD by national stakeholders.^45 46^ Weak national institutions and country governance and leadership deficits may exacerbate this power imbalance.^45 46^ In addition to a donor country’s international aid agenda, their commercial and economic interests may pervade their views on the PD subject matter and ultimately on how the PD is conducted,^53^ leading to mistrust and even disillusionment within national institutions, subsequently weakening the dialogue.^47^ These negative experiences, coupled with the imbalance of power in favour of donors, could lead to a real risk of recipient country disengagement from PD. Some authors highlight the need for strong negotiation skills, especially on the part of donors, to prevent decisions from being perceived as imposed from the outside.^48 53^ This power imbalance can also slow down the overall PD process if there are no common interests at stake.^46 63^ It can also lead to a PD process that does not lead to change and maintains the status quo.^59^ The donor-recipient power imbalance is also cited as a cause of recipient country decision-makers’ agreeing to proposals without having the capacity or real intention to implement them.^55^

### The practicalities of implementing policy dialogue as a governance mechanism

This section refers to the literature that views PD as a mode of governance or a negotiating instrument. This literature differs significantly from the literature where PD is conceived as a knowledge exchange and translation platform. It more often addresses processual challenges using policy analysis or development studies frameworks or concepts. As such, it provides useful insights on PD as a mechanism to foster collaborative governance.

The literature that refers to PD as a mode of governance identifies sustaining participation as a key implementation challenge. Motivating and maintaining stakeholder participation is driven by a belief or expectation that engagement in the PD process can lead to change or improvement.^48^ If no perceptible change happens, or the status quo remains, a negative perception of both PD and national institutional implementation capacity can ensue.^61^

It is difficult for a PD process to be credible if participants have no experience in the technical area.^48^ Participation could also be hindered by a lack of rules that institutionalise PD practice and stakeholder involvement.^44 48^ According to one author, the more PD is institutionalised, the more positive the results are.^41^ The literature demonstrated that a problem, when highly politicised, could encourage the emergence of ad hoc spaces for dialogue, which could gradually become institutionalised by virtue of repeatedly taking place at key moments in the policy development process.^40^ To encourage participation by civil society and hard-to-reach population groups,^39^ PD could be organised by several small groups (potentially in a focus group format) whose decisions feed into a more strategic-level PD.^41 61^ Another option would be to organise smaller PDs at decentralised administrative levels where policy operationalisation happens.^48^

As a negotiating instrument, PD is seen as a process where the players have a strategic interest and stake in the PD topic. Most of the literature, particularly the grey literature, therefore focuses on strategies for influencing PD, particularly in the context of development aid. To create the right conditions for PD, development aid stakeholders should be aware of the reasons for participating in PD and what they can expect from it.^28 54^ Moreover, the methodology seems less crucial than the choice of the PD approach, which can be a technical or diplomatic one, depending on the nature and sensitivity of the PD subject. The technocratic nature of PD may, therefore, become a problem for three main reasons:

1. The proposed solutions may not take account of what is socially or politically acceptable.
2. International experts may make incorrect assessments.^60^
3. Technical assistance may be seen as support for political regimes perceived to be illegitimate.^51^

At a global level, the different donors may not agree with each other and favour different approaches to development aid and PD.^51^ Indeed, different donors can sometimes have antithetical, even irreconcilable positions, yet all seek to bring aid to the same country, leading to general resistance from recipient institutions.^53^ This may explain why donors first try to coordinate and harmonise their messages among themselves, which sometimes gives national institutions the impression that they are working together to their detriment.

## Discussion

### Lessons learnt

This scoping study reveals the polysemy of the notion of policy dialogue. PD may be an organised, specific activity, which has a specific purpose, focusing on evidence and knowledge exchange and translation. It may also be a mode of governance, a modus operandi for public policy, a process for developing public policies.^64^ PD may finally be a strategic way of influencing public sector activities by facilitating discussions and negotiations between stakeholders, and particularly between donors and partner countries in the context of development aid. Some authors borrow from several of these approaches to define or study PD.^65^

This study also highlights the need for adequate resources and funding to organise and inform PD. It exposes three conditions to foster continued stakeholder engagement: a transparent and institutionalised process, a shared understanding of the goals of PD and an approach that fits the intended goals. Critical factors of success include: (a) stakeholders remain engaged during all relevant PD stages, (b) they bring institutional or group perspectives to the discussions and represent the collective voice of their organisation/group, (c) they are interested and have a stake in the topic.

In this paper, we highlight development aid as a distinct context where specific challenges occur in PD. Unequal power relations between stakeholders, weak country-level organisational or technical capacities to support or contribute constructively to PD and PD that is controlled by technocrats may lead to ineffective PD in aid-dependent settings.

### Different stakeholders, different objectives

Our analysis also shows that the different notions of PD reflect the different perspectives and objectives of each stakeholder group. For the research community, PD offers a way of bringing evidence into the policy development process. Their aims are to inform, raise awareness and discuss the issues raised by research results. These very objectives are at the heart of knowledge exchange and translation strategies.^66^ For institutional decision-makers, PD is useful in securing buy-in for policy decisions, understanding other stakeholders’ viewpoints and bringing in lay knowledge and lived experiences to inform realistic decisions.^67^ For civil society, PD is an opportunity for voices to speak up and for populations and communities to influence decision-making.^14 16^ The aim is, therefore, to flag policy issues, bring in evidence or advocate for alternatives or guide decision-makers towards new solutions. In the context of development aid, international donors see PD as a way of streamlining the policy process and contributing to good governance.^68^ More specifically, their goal is to support and prioritise reforms while leveraging PD as a mechanism for cooperation with national institutions.

### Addressing policy dialogue challenges

One of the main challenges raised by the scoping study is the lack of stakeholder capacity for PD, be it government cadres, civil society actors or other stakeholders. Government cadres generally hail from a medico-technical background and are not trained in collaborative governance, which requires abilities to build consensus, deal with opposing views and convey participants to dialogue effectively. As the role of Ministries of Health changes from service delivery organisations to stewards of the health system,^69^ they need to develop such new skill sets. As for civil society actors, they may lack the skills or experience to analyse or understand scientific evidence, or to partner with evidence generators in order to advocate their position in discussions on policy options.^70 71^ Ultimately, when stakeholders do not have the necessary skills to initiate or engage effectively in PD, PD remains an empty shell and may reinforce power imbalances. Such PD is less likely to lead to acceptable health policies and plans, jeopardising their implementation. It is hence urgent to strengthen participant’s capacities.

Ensuring funding for PD, especially in LMIC, is also mentioned as a prerequisite to effective PD. Although fundamental, this issue appears overlooked in the literature where PD is viewed as a mode of governance or negotiating instrument, with some exceptions.^42 56 72^ Such funding is yet crucial to organise concertation processes, build the capacity of conveners and facilitators and train staff to mobilise and synthesise evidence in a policy-relevant way. These activities require steady and predictable monies to ensure sustainability in PD efforts.

Addressing these challenges requires general recognition from the global health community of the criticality of putting attention and resources to strengthening health governance at national levels while making inroads in other health systems areas. This combined action is necessary to craft health systems that respond to the needs of the most vulnerable.^4^ To the knowledge of the authors, PD has not yet been the object of an investment case that might further enlighten decision-makers, civil society and funders about its importance. The UHC Partnership run by WHO, in collaboration with a variety of partners, aims to provide initial funding and technical expertise to the Ministry of Health in LMIC to bolster PD. Evaluations of the UHC Partnership show that PD does not require substantial financial support to be sustained and effective. Instead, it needs low but constant funding. A high level of technical expertise is, however, essential to understand the context, analyse evidence and support PD.^73–76^ Approaches such as the UHC Partnership may serve as an example to address capacity shortfalls and guarantee funding to foster PD and strengthen health system governance.

### Limitations

The main limitation of this review is that we narrowed our search to two keywords, being ‘policy dialogue’ and ‘multistakeholder/multistakeholder dialogue’. Other research areas may label PD differently or interpret it differently, particularly in terms of citizen participation, giving rise to many forms of dialogue, for example, science-policy dialogue, deliberative dialogue, social dialogue or collaborative forum. Such a diversity of terms explains why we focused on English literature, regrettably excluding potentially relevant papers in other languages, which is another limitation of this review. We deliberately excluded other terminologies because our aim was not to provide a classification of governance collaborative tools or types of dialogue. We instead sought to unveil the nuances in defining and operationalising PD and give an overview of the state of knowledge about PD. The vague outlines of PD also made it difficult to apply inclusion criteria, even more so as its definition was honed over the course of the analysis. Pieces of literature may therefore have been overlooked during the selection process. In addition, the lessons learnt highlighted by this study need to be qualified for three main reasons. First, the papers on PD in the grey literature have not systematically used scientific methodology. Second, if studies are commissioned or conducted by organisations that support PD, there is a desirability bias. Finally, a scoping study does not aim to explore how robust the evidence is, a task rendered particularly difficult, if not futile, by the range of disciplinary approaches and conceptual frameworks used to study the multiple facets of PD.^65^ This study therefore provides a starting point for a more comprehensive and in-depth analysis of the literature, such as a realist^77^ or meta-narrative^78^ review, focusing on new mechanisms for participatory and collaborative governance already identified elsewhere.^79 80^

## Conclusion

The objective of this scoping study was to clarify the concept of PD and understand potential challenges in implementing and conducting PD as a collaborative governance tool. This review highlights key ingredients and conditions or contextual factors that may affect PD implementation, sustainability and outcomes, as well as the participation of stakeholders in PD. As such, it is an additional building block in the research on collaborative governance of health in LMIC,^7^ and a step towards improved clarity among health systems researchers and professionals.

Policy dialogue, which should not be confused with political dialogue, could prove to be relevant in strengthening multistakeholder governance. Among the many critical conditions needed so that PD becomes a genuinely transformative tool in LMIC, two should be given priority: first, PD must be supported by public agencies that are empowered. Second, global health actors, who see it as an opportunity to influence public action, must engage in good faith, particularly where there is weak institutional governance and the balance of power is in their favour.
